# Gossypol Suppresses Growth of Temozolomide-Resistant Glioblastoma Tumor Spheres

**DOI:** 10.3390/biom9100595

**Published:** 2019-10-10

**Authors:** Hee Yeon Kim, Byung Il Lee, Ji Hoon Jeon, Dong Keon Kim, Seok-Gu Kang, Jin-Kyoung Shim, Soo Youl Kim, Sang Won Kang, Hyonchol Jang

**Affiliations:** 1 Division of Cancer Biology, Research Institute, National Cancer Center, Goyang 10408, Korea; 74790@ncc.re.kr (H.Y.K.); jh6107@hanmail.net (J.H.J.); kimdk@ncc.re.kr (D.K.K.); kimsooyoul@gmail.com (S.Y.K.); 2 Department of Life Science, Ewha Womans University, Seoul 03760, Korea; Kangsw@ewha.ac.kr; 3 Division of Precision Medicine, Research Institute, National Cancer Center, Goyang 10408, Korea; bilee@ncc.re.kr; 4 Department of Cancer Biomedical Science, National Cancer Center Graduate School of Cancer Science and Policy, Goyang 10408, Korea; 5 Department of Neurosurgery, Brain Tumor Center, Severance Hospital, Yonsei University College of Medicine, Seoul 03722, Korea; seokgu9@gmail.com (S.-G.K.); nanjk2@yuhs.ac (J.-K.S.)

**Keywords:** Bcl2, dehydrogenase, glioblastoma, gossypol, temozolomide resistance

## Abstract

Temozolomide is the current first-line treatment for glioblastoma patients but, because many patients are resistant to it, there is an urgent need to develop antitumor agents to treat temozolomide-resistant glioblastoma. Gossypol, a natural polyphenolic compound, has been studied as a monotherapy or combination therapy for the treatment of glioblastoma. The combination of gossypol and temozolomide has been shown to inhibit glioblastoma, but it is not clear yet whether gossypol alone can suppress temozolomide-resistant glioblastoma. We find that gossypol suppresses the growth of temozolomide-resistant glioblastoma cells in both tumor sphere and adherent culture conditions, with tumor spheres showing the greatest sensitivity. Molecular docking and binding energy calculations show that gossypol has a similar affinity to the Bcl2 (B-cell lymphoma 2) family of proteins and several dehydrogenases. Gossypol reduces mitochondrial membrane potential and cellular ATP levels before cell death, which suggests that gossypol inhibits several dehydrogenases in the cell’s metabolic pathway. Treatment with a Bcl2 inhibitor does not fully explain the effect of gossypol on glioblastoma. Overall, this study demonstrates that gossypol can suppress temozolomide-resistant glioblastoma and will be helpful for the refinement of gossypol treatments by elucidating some of the molecular mechanisms of gossypol in glioblastoma.

## 1. Introduction

Glioblastoma, the most aggressive primary malignant brain tumor with a median survival of 15 months, is currently treated with a first-line combination of surgery, radiation therapy, and temozolomide (TMZ) [1]. Although TMZ improves the median survival of patients by only two- and one-half months compared to radiotherapy alone [2], no other drugs have been developed that can improve patient survival better than TMZ [3]. Its main limitation impeding patient survival is that over 50% of TMZ-treated patients do not respond to TMZ [4]. Thus, there is an urgent need to find novel candidates to treat TMZ-resistant glioblastoma.

Gossypol is a natural polyphenolic compound extracted from cotton plants and its R-(−)-enantiomer also is known as AT-101 (or (−)-gossypol) [5,6,7]. Gossypol has been studied for its potential as an antitumor agent in various cancers, including breast cancer [8,9], lung cancer [10,11], colon cancer [12], bladder cancer [13], and pancreatic cancer [14]. Additionally, many clinical trials using gossypol to treat cancer have been conducted or are in progress (Table 1). Regarding glioblastoma, a number of studies and clinical trials have been conducted that use gossypol alone or in combination for the treatment of patients. Gossypol induced cell death in glioblastoma cell lines [15,16] and showed a low, but measurable, response rate in heavily pretreated, poor-prognosis patients with recurrent glioblastoma [17]. The combination of gossypol with TMZ [18,19,20], phenformin [21], or arsenic trioxide [22] showed significant suppression of glioblastoma cell lines or patient-derived glioblastoma tumor spheres (TSs). It is still unclear, however, whether gossypol effectively inhibits TMZ-resistant glioblastoma so further studies are needed before gossypol can be used in clinical practice.

Gossypol inhibits various proteins, including the Bcl2 (B-cell lymphoma 2) family of proteins [6,23,24], lactate dehydrogenases [25,26], malate dehydrogenases [25,26], isocitrate dehydrogenases [26], glyceraldehyde dehydrogenases [26], aldehyde dehydrogenases [27,28], and APEX1 (Apurinic/Apyrimidinic endodeoxyribonuclease 1) [29]. Despite having many targets, most clinical trials attempting to use gossypol as an anticancer agent have considered gossypol to be an inhibitor of the Bcl2 family of proteins (Table 1). Considering the case of glioblastoma, it appears that the main targets of gossypol are the Bcl2 family proteins [15,16,19], several dehydrogenases [17], and aldehyde dehydrogenase [21]. Since understanding the molecular mechanism is critical for drug improvement, it is important to clarify the main targets of gossypol in glioblastoma.

We show that gossypol ((±)-gossypol, a racemic mixture of R and S enantiomer) effectively suppresses the growth of TMZ-resistant patient-derived glioblastoma cells and that both the Bcl2 family proteins and various dehydrogenases are important targets of gossypol in glioblastoma.

## 2. Materials and Methods

### 2.1. Cell Culture and Reagents

Cultures of patient-derived TS13-18 and TS13-20 cells has been described previously [30]. Briefly, both cell lines were established directly from male patients with *IDH1* (Isocitrate dehydrogenase 1) wild-type primary World Health Organization (WHO) grade 4 glioblastomata. While the *MGMT* (*O*^6^-alkylguanine DNA alkyltransferase) gene promoter in TS13-18 was unmethylated, the same promoter in TS13-20 was methylated. Glioblastoma TS cells and their differentiated counterparts were cultured as previously described [30]. Briefly, TS cells were cultured in growth media at 37 °C in a 5% CO_2_ humidified incubator. The differentiated counterparts were cultured under the same conditions but supplemented with 10% heat-inactivated fetal bovine serum (FBS; #SH30084.03; HyClone, Logan, UT, USA). Growth media consisted of DMEM/F-12 (#10-0900cv; HyClone) supplemented with 1× B27 (#17504-044; Invitrogen, San Diego, CA, USA), 20 ng/mL basic fibroblast growth factor (#E0291; Sigma–Aldrich, St. Louis, MO, USA), 20 ng/mL epidermal growth factor (#E9644; Sigma–Aldrich), and 1% penicillin-streptomycin (#15140-122; Invitrogen). Gossypol (#G8761) and TMZ (#T2577) were purchased from Sigma–Aldrich and ABT-263 (#A3007) was obtained from APExBIO (Boston, MA, USA).

### 2.2. Limiting Dilution Assays

Cells were dissociated as single cells using Accutase (#561527; BD Biosciences, San Jose, CA, USA) and were serially diluted from 50 to 5 cells per well in 16 replicates into 96-well clear flat bottom ultra-low binding microplates (#3474; Corning, Corning, NY, USA) in growth media with or without gossypol or TMZ. Following two weeks, empty wells were counted. Extreme limiting dilution analysis was performed using the software ELDA (Extreme limiting dilution analysis) [31].

### 2.3. Detection of Apoptosis

Cells were treated with gossypol (10 μM) for the indicated periods. Then, 1 × 10^5^ cells were stained with an annexin V-FITC/propidium iodide (PI) detection kit (#LS-02-100; BioBud, Seongnam, Korea). Apoptotic cells were analyzed using a FACSVerse flow cytometer (BD Biosciences) in the Flow Cytometry Core Facility (National Cancer Center, Goyang, South Korea).

### 2.4. Western Blot

Western blot was performed as previously described [32]. Anti-caspase-3 (#9662) and anti-PARP (Poly (ADP-ribose) polymerase; #9542) antibodies were purchased from Cell Signaling Technology (Danvers, MA, USA). The Anti-β-actin (#A2228) antibody was purchased from Sigma–Aldrich.

### 2.5. Binding Energy Calculation

Docking models for various target protein structures and the gossypol complex were generated using the Glide-SP module from the Schrödinger Suite software package (version 2016-1; Schrödinger, LLC, New York, NY, USA) and their binding free energies were calculated using the Prime MM/GBSA (Molecular Mechanics–Generalized Born Surface Area) module. The binding free energy ΔG_bind_ was calculated using the equation ΔG_bind_ = G_complex_ − (G_protein_ + G_ligand_). Regarding IDH1 and IDH2, the protein–ligand binding energy was calculated for two different ligand binding pockets (inhibitor or NADP binding sites).

### 2.6. Image-Based Quantification of Sphere and Cell Numbers

Images of spheres and differentiated cells were obtained and quantified as previously described [30,33]. Briefly, images were obtained using a Cyation-3 cell imaging multimode microplate reader with a ×4 objective (Bio-Tek, Winooski, VT, USA). Considering the case of differentiated cells, cells were incubated with DAPI (2 μg / ml) for 20 min at room temperature before obtaining images. Images were analyzed using the ImageJ program. Colonies >10 μm in diameter were considered sphere cells.

### 2.7. Measurement of Mitochondrial Membrane Potential 

Mitochondrial membrane potential (MMP) was measured by flowcytometry as previously described [33] using the TMRE-Mitochondrial Membrane Potential Assay Kit (#ab113852; Abcam, Cambridge, UK). Briefly, cells were dissociated to single cells using trypsin/EDTA and incubated with TMRE (100 nM), then analyzed by FACSVerse flow cytometry (BD Biosciences). 

### 2.8. Measurement of ATP Level

The ATP level was measured using the CellTiter-Glo Luminescent Cell Viability Assay kit (#G7572; Promega, Madison, WI, USA) as per the manufacturer’s instructions, as previously described [33].

### 2.9. Cell Viability Assay

Cell viability was determined using the MTT (3-(4,5-dimethylthiazol-2-yl)-2,5-diphenyltetrazolium bromide) assay (#0793; AMRESCO, Solon, OH, USA) according to the manufacturer’s instructions. Briefly, an MTT solution was added to cells that had been treated with or without gossypol (10 μM) for three days, and then incubated at 37 °C to form purple formazan. Formazan was dissolved with dimethyl sulfoxide and absorbance was measured at 570 nm using a microplate reader (#89429-538; Molecular Devices, San Jose, CA, USA).

### 2.10. Statistical Analysis

Statistical analyses were performed as previously reported [33]. Data were presented as the mean ± standard deviation, and *p*-values were calculated using the Student’s *t*-test. All data were representative of at least three separate experiments.

## 3. Results

### 3.1. Gossypol Suppresses Growth of Temozolomide-Resistant Glioblastoma Cells

**Figure 1:**
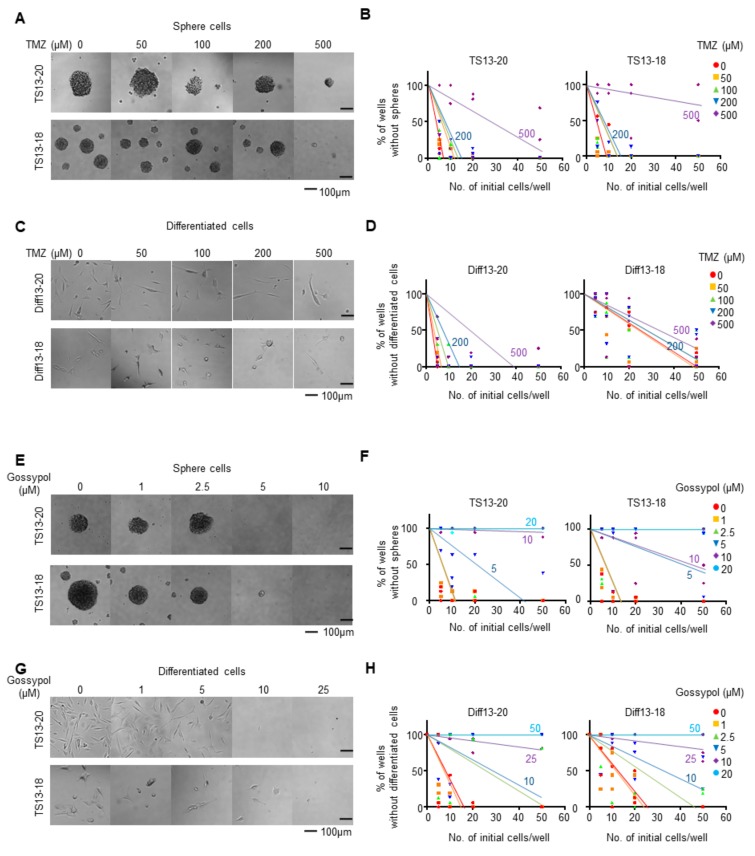
Gossypol suppresses temozolomide-resistant glioblastoma cells. (**A**) Tumor spheres (TS)13-20 and TS13-18 glioblastoma sphere cells were treated with the indicated concentrations of temozolomide (TMZ) for 14 days. Bright field images of the cells were taken and a representative image of three independent experiments is shown. (**B**) Limiting dilution assays were performed for TS13-20 and TS13-18 cells treated with the indicated concentrations of TMZ. (**C**) TS13-20 and TS13-18 glioblastoma sphere cells were differentiated by the addition of serum for seven days and named Diff13-20 and Diff13-18, respectively. Differentiated cells were treated with TMZ for 14 days and bright field images of the cells are shown. (**D**) Limiting dilution assays were performed for Diff13-20 and Diff13-18 cells treated with the indicated concentrations of TMZ. (**E**–**H**) The same experiments as in (**A**–**D**) were performed using gossypol rather than TMZ.

We first tested whether glioblastoma TS cells, TS13-18 (with unmethylated *MGMT* gene promoter) and TS13-20 (with methylated *MGMT* gene promoter), were responsive to TMZ. Following two weeks of TMZ treatment up to a concentration of 200 µM, there were no apparent changes in TS size and number (Figure 1A). A limiting dilution assay also showed that TMZ treatment up to a concentration of 200 µM did not affect TS formation, and even 500 µM of TMZ did not completely block TS formation (Figure 1B). Since stemness of glioblastoma is one of the major causes of glioblastoma resistance to TMZ [34], we tested whether resistance to TMZ was reduced by differentiation of TS cells. Serum addition for seven days caused TS cells to adhere to the culture plate and reduce stemness [30]. We named these cells Diff13-20 and Diff13-18, respectively. Differentiated cells showed no apparent cell death from TMZ treatment up to a concentration of 100 µM (Figure 1C). A limiting dilution assay showed no significant change from TMZ treatment up to 200 µM, and even 500 µM of TMZ did not completely kill differentiated cells (Figure 1D). These data showed that TS13-18 and TS13-20 cells, which were established directly from male patients with IDH1 wild-type primary WHO grade 4 glioblastomata, were resistant to TMZ treatment. 

Next, we investigated whether the growth of TMZ-resistant glioblastoma cells could be inhibited by gossypol. A two-week treatment of 5 µM gossypol clearly inhibited TS size and number (Figure 1E). A limiting dilution assay also showed that 5 µM gossypol significantly reduced TS formation and that TS formation was completely blocked by treatment with 10 µM gossypol for TS13-20 and 20 µM gossypol for TS13-18 (Figure 1F). The growth of differentiated glioblastoma cells also was inhibited by gossypol, but at a slightly higher concentration than TS; Diff13-20 cells were inhibited by gossypol at 10 µM and Diff13-18 cells were inhibited at 25 µM (Figure 1G). A limiting dilution assay showed that 25–50 µM gossypol was required to completely inhibit differentiated cells (Figure 1H). These data suggest that gossypol can inhibit effectively the growth of TMZ-resistant glioblastoma cells, and that glioblastoma TS cells are more sensitive to gossypol than their differentiated counterparts.

### 3.2. Gossypol Induces Apoptosis in Both Glioblastoma Tumor Spheres and Differentiated Cells

**Figure 2:**
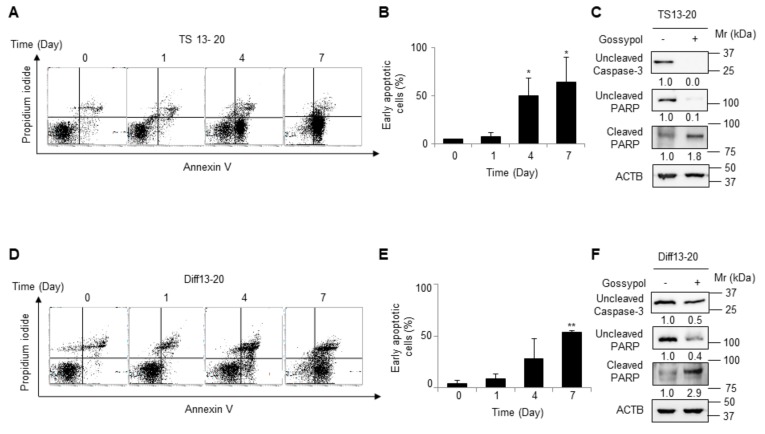
Gossypol causes apoptosis in both glioblastoma tumor sphere and differentiated cells. (**A**,**B**) TS13-20 cells were treated with gossypol (10 μM) for the indicated periods. Then, cells were stained using an annexin V kit (BD Biosciences). The percentages of early apoptotic cells were analyzed by flow cytometry (FACSVerse; BD Biosciences) and are indicated as the mean ± standard deviation (*n* = 3). * *p* < 0.05 relative to day 0. (**C**) TS13-20 cells were treated with gossypol (10 μM) for four days. Protein levels of uncleaved caspase-3 and PARP, and cleaved PARP were analyzed by western blot. β-Actin (ACTB) was used as a loading control. (**D**–**F**) The same experiments as in (**A**–**C**) were performed using Diff13-20 instead of TS13-20. **p <0.01 relative to day 0. The numbers below the blot images in (**C**,**F**) indicate the relative expression normalized by β-Actin.

To determine the mechanism of glioblastoma cell death caused by gossypol, we checked whether gossypol treatment induced apoptosis of glioblastoma cells. Gossypol-treated TS13-20 cells showed upregulation of early apoptosis in a time-dependent manner as determined by flow cytometry of annexin V-positive and PI-negative fractions (Figure 2A,B). Gossypol-treated TS13-20 cells showed decreased uncleaved caspase-3 and PARP and increased cleaved PARP, which clearly showed that gossypol induced apoptosis of glioblastoma TS (Figure 2C). Gossypol-treated Diff13-20 cells showed similar phenomena (Figure 2D–F). These results suggest that gossypol induces apoptosis of glioblastoma in both three-dimensional TS cell culture and two-dimensional adherent cell culture.

### 3.3. The Mode of Action of Gossypol in Glioblastoma

**Figure 3:**
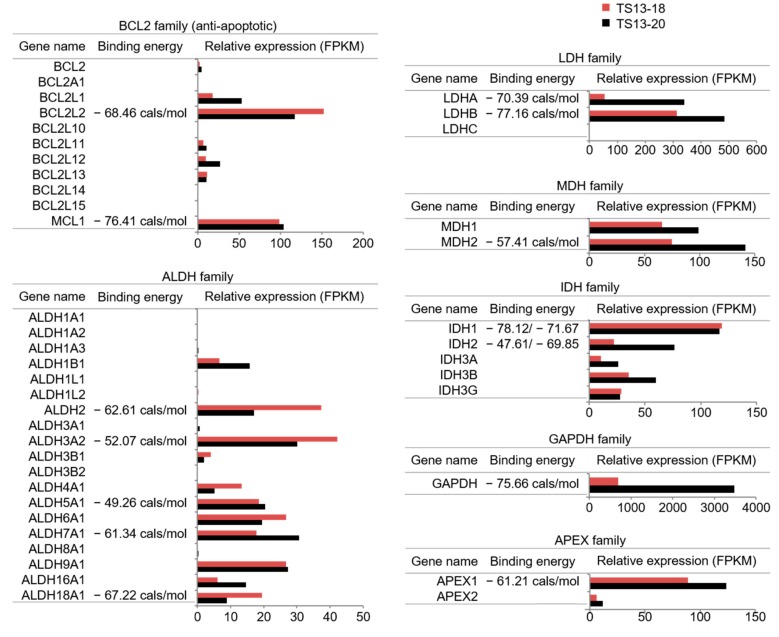
Gossypol can bind to various dehydrogenases as well as to BCL2 family proteins. The expression levels of potential gossypol target genes were obtained from previously published RNA-sequencing data of TS13-2- and TS13-18 [30]. Binding energies were calculated for docking structures of gossypol–BCL2 family (BCL2L2 and MCL1), gossypol–dehydrogenases (ALDH, LDH, MDH, IDH, and GAPDH), and gossypol–APEX using the MM/GBSA method. The Protein Data Bank entries used for the docking and MM/GBSA binding energy calculations were 4CIM (BCL2L2), 4OQ5 (MCL1), 1O04 (ALDH2), 4QGK (ALDH3A2), 2W8N (ALDH5A1), 4ZVX (ALDH7A1), 2H5G (ALDH18A1), 4ZVV (LDHA) 1I0Z (LDHB), 2DFD (MDH2), 5DE1 (IDH1), 4JA8 (IDH2), 1U8F (GAPDH), and 5DFI (APEX1), respectively. FPKM; Fragments per kilobase of transcript per million.

Although gossypol has been shown to interact with various proteins, including the Bcl2 family of proteins [6] and dehydrogenases [25,26,27,28], several studies on the role of gossypol in glioblastoma assumed Bcl2 family proteins to be the main targets of gossypol [15,16,19]. Additionally, most clinical trials of gossypol as a cancer treatment have made the same assumption (Table 1). To clarify the mode of action of gossypol in glioblastoma, we first selected genes among the potential targets of gossypol that were highly expressed in glioblastoma using previously reported TS13-20 and TS13-18 RNA-sequencing results [30]. Potential targets of gossypol in glioblastoma TS were BCL2L2 (Bcl2 like 2), MCL1 (Myeloid cell leukemia 1), APEX, and several dehydrogenases (Figure 3). Regarding the selected genes, we performed an in silico calculation to estimate how strongly the proteins could bind to gossypol. Molecular docking and a combined MM/GBSA binding energy calculation showed that gossypol had a similar binding affinity for the Bcl2 family proteins and several dehydrogenases (Figure 3 and Supplementary Figure S1). 

**Figure 4:**
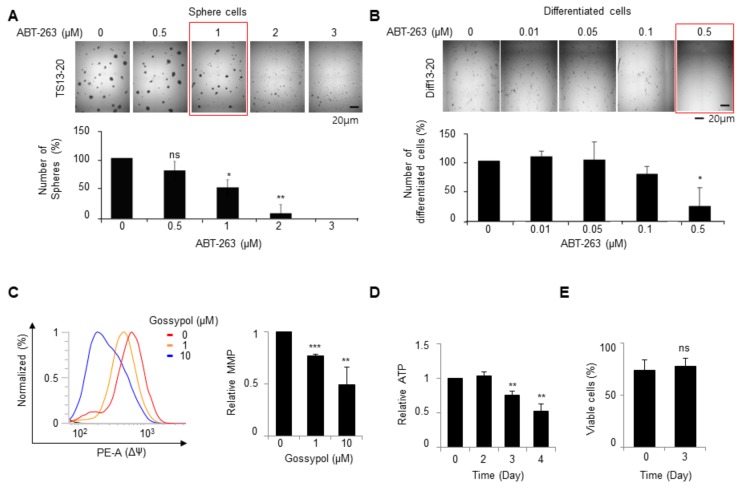
The function of gossypol as a BCL2 family inhibitor does not explain fully the mode of action of gossypol in glioblastoma. (**A**,**B**) TS13-20 and Diff13-20 cells were treated with the indicated concentrations of ABT263, a BCL2 inhibitor, for 7 days. A bright field image of the cells was taken using a Cytation 3 microplate reader (BioTek). The number of spheres or cells were quantified as described in Section 2 (*n* = 3). ** *p* < 0.01, * *p* < 0.05, NS (not significant) *p* > 0.05 relative to the number of cells in the ABT-263 untreated condition. (**C**) TS13-20 cells were treated with the indicated concentrations of gossypol for 24 h. The mitochondrial membrane potential (MMP) was investigated by tetramethylrhodamine ethyl ester staining followed by flow cytometry (FACSVerse; BD Biosciences). Values indicate the mean ± standard deviation (*n* = 3). ** *p* < 0.01, *** *p* < 0.001 relative to the gossypol untreated condition. (**D**) Diff13-20 cells were treated with gossypol (10 μM) for the indicated periods and cellular ATP levels were determined using an ATP assay kit. Values indicate the mean ± standard deviation (*n* = 3). ** *p* < 0.01. (**E**) Diff13-20 cells were treated with gossypol (10 μM) for three days, and cell viability was determined using the MTT assay. Values indicate the mean ± standard deviation (*n* = 3). NS *p* > 0.05.

Treatment with ABT-263, an inhibitor of Bcl2 family proteins, resulted in the inhibition of both TS13-20 and Diff13-20 (Figure 4A,B). However, Diff13-20 was more sensitive to ABT-263 than TS13-20 (Figure 4A,B), which was inconsistent with the finding that TS13-20 was more sensitive to gossypol than Diff13-20 (Figure 1E–H). Additionally, gossypol treatment for 24 h significantly reduced MMP in TS13-20 cells (Figure 4C) and decreased cellular ATP levels before cell death (Figure 4D,E). This finding indicates that gossypol regulates mitochondrial function in glioblastoma cells. Considering the results of the binding energy calculations, it is likely that gossypol regulates MMP and ATP levels by inhibiting important dehydrogenases in the cell’s metabolic pathway. 

Overall, our study demonstrates that gossypol can suppress the growth of TMZ-resistant glioblastoma cells in both three-dimensional TS and two-dimensional adherent culture conditions, and the mode of action of gossypol in glioblastoma is not restricted to the inhibition of Bcl2 family proteins.

## 4. Discussion

TMZ resistance is a major obstacle to improving the outcomes in patients with glioblastoma. The primary mechanisms underlying TMZ resistance are thought to be an overexpression of O^6^-methylguanine methyltransferase (*MGMT*) and a lack of a DNA repair pathway [4]. Methylation of the *MGMT* gene promoter has been demonstrated to be the strongest prognostic marker for TMZ susceptibility, and the added benefit of TMZ chemotherapy appears to be largely restricted to this subgroup [35]. The *MGMT* gene promoters in TS13-18 and TS13-20, however, were unmethylated and methylated, respectively, and both were resistant to TMZ treatment (Figure 1A–D). Thus, the two cell types used in this study represented TMZ-resistant glioblastoma regardless of *MGMT* status. 

Cancer stemness is another mechanism associated with TMZ resistance [36]; however, the TMZ resistance of both cell types was too high to identify whether the TS or differentiated cells were more resistant. Gossypol inhibited both TS13-18 and TS13-20, but somewhat higher concentrations were required for the differentiated cells (Figure 1E–H). Since TS formation is affected by both cancer stemness and cancer cell viability, there is a limit to linking reduced TS formation to reduction of cancer stemness. Additionally, TMZ has been reported to induce dormant stem cell-like cells and gossypol (AT-101) has shown a high cytotoxic effect against glioblastoma cells with low TMZ effect when combined with TMZ [19]. Given this report and the results of this study, gossypol is likely to reduce cancer stemness, but more stringent evidence is required. 

The mechanism of gossypol-induced cell death is controversial. Gossypol caused apoptosis in patient-derived TMZ-resistant glioblastoma, as determined by annexin V staining, followed by flowcytometry and cleavage of caspase-3 and PARP by western blot (Figure 2). Consistent with our results, gossypol (AT-101) induced apoptosis in U251 and U343 cell lines [37] and in GS-5 glioma stem cells [22]; however, gossypol (AT-101) induced autophagic cell death in U343 [15,38] and enhanced radiation induced autophagy in the U87 MG cell line [39]. Given that glioblastoma stem cells generally are resistant to chemotherapy [40] and autophagy is associated with stem-like phenotypes [41], further research is needed to clearly determine whether gossypol can reduce glioblastoma stemness. 

According to several completed phase 1/2 clinical trials, gossypol does not cause serious side effects in various types of cancer patients (Table 1), suggesting that gossypol will be clinically available in the near future. Gossypol monotherapy and combination therapy, however, have shown marginal effects in various clinical trials (Table 1), suggesting that improvements in gossypol or new combinations are needed. To achieve these goals, it is necessary to clarify the molecular target of gossypol when it shows anticancer activity. Although most clinical trials have considered gossypol’s main target to be the Bcl2 family of proteins (Table 1), gossypol also has been shown to inhibit various dehydrogenases. Granting there is solid evidence that gossypol inhibits individual proteins for several target proteins, it is unclear which targets are inhibited more sensitively and which ones are most associated with its anticancer effects. Calculating the binding energy of gossypol to its targets after molecular docking showed that gossypol likely binds Bcl2 family proteins and several dehydrogenases with similar affinity. ABT-263, an inhibitor of Bcl2 family proteins, did not reproduce gossypol’s effects in TMZ-resistant glioblastoma. These results suggest that the role of gossypol as an inhibitor of various dehydrogenases should be considered when improving gossypol and designing combination therapies, and more intensive studies of its molecular mechanisms are needed. 

To conclude, gossypol can suppress the growth of TMZ-resistant glioblastoma, and its role as an inhibitor against various proteins should be considered fully for the improvement of gossypol monotherapy and combination therapies.

## Figures and Tables

**Table 1:** Completed and ongoing clinical trials of gossypol to treat cancer.

Status	Study Title	Phase	Main Targets	Tumor Type
Completed	Gossypol in Treating Patients with Progressive or Recurrent Glioblastoma Multiforme	Phase 2	Bcl-2 family	Glioblastoma
Completed	Gossypol (AT-101) and Temozolomide With or Without Radiation Therapy in Treating Patients with Newly Diagnosed Glioblastoma Multiforme	Phase 1	Bcl-2 family	Glioblastoma
Completed	Gossypol Acetic Acid in Treating Patients with Recurrent, Metastatic, or Primary Adrenocortical Cancer that Cannot be Removed by Surgery	Phase 2	unclear	Adrenocortical carcinoma
Completed	R-(−)-Gossypol Acetic Acid, Cisplatin, and Etoposide in Treating Patients with Advanced Solid Tumors or Extensive-Stage Small-Cell Lung Cancer	Phase 1	Bcl-2 family	Small-cell lung cancer, advanced solid tumor
Unknown	Gossypol Combined with Docetaxel and Cisplatin Scheme in Advanced Non-Small-Cell Lung Cancers with APE1 High-Expression	Phase 3	APE1	Non-small-cell lung cancer
Withdrawn	Tarceva and AT-101 for Patients with Advanced Non-Small-Cell Lung Cancer	Phase 1	Bcl-2 family	Non-small-cell lung cancer
Suspended	R-(−)-Gossypol Acetic Acid with Lenalidomide and Dexamethasone in Treating Patients with Relapsed Symptomatic Multiple Myeloma	Phase1/2	Bcl-2 family	Recurrent plasma cell myeloma
Completed	R-(−)-Gossypol Acetic Acid in Treating Patients with Recurrent Extensive-Stage Small-Cell Lung Cancer	Phase 2	Bcl-2 family	Small-cell lung cancer
Terminated	Erlotinib and AT-101 in Advanced Non-Small Cell Lung Cancer (NSCLC) Patients with Epidermal Growth Factor Receptor (EGFR) Activating Mutations	Phase 2	Bcl-2 family	Non-small-cell lung cancer
Active, not recruiting	Lenalidomide and AT-101 in Treating Patients with Relapsed B-Cell Chronic Lymphocytic Leukemia	Phase1/2	Bcl-2 family	Chronic lymphocytic leukemia
Completed	Phase 2 Safety and Efficacy Study of AT-101 in Combination with Rituximab in Patients with Chronic Lymphocytic Leukemia	Phase 2	Bcl-2 family	Chronic lymphocytic leukemia
Completed	R-(−)-Gossypol and Androgen Ablation Therapy in Treating Patients with Newly Diagnosed Metastatic Prostate Cancer	Phase 2	Bcl-2 family	Prostate cancer
Completed	Safety and Efficacy Study of AT-101 in Combination with Docetaxel and Prednisone in Men With HRPC	Phase1/2	Bcl-2 family	Prostate cancer
Terminated	An Open-Label, Single-Center, Phase 1/ 2 Study of Chemoradiotherapy and AT-101 in Patients with Locally Advanced Esophageal or Gastroesophageal Junction Cancer	Phase1/2	Bcl-2 family	Esophageal or Gastroesophageal junction cancer
Completed	Gossypol, Paclitaxel, and Carboplatin in Treating Patients with Solid Tumors That Are Metastatic or Cannot Be Removed by Surgery	Phase 1	unclear	Lymphoma
Completed	A Randomized Phase 2 Study of AT-101 in Combination with Docetaxel in Relapsed/Refractory Non-Small-Cell Lung Cancer	Phase 2	Bcl-2 family	Non-small-cell lung cancer
Completed	A Study Comparing AT-101 in Combination with Docetaxel and Prednisone Versus Docetaxel and Prednisone in Men with Chemotherapy-Naive Metastatic Hormone Refractory Prostate Cancer (HRPC)	Phase 2	Bcl-2 family	Hormone refractory prostate cancer
Completed	Study of AT-101 in Combination with Topotecan in Relapsed/Refractory Small-Cell Lung Cancer	Phase1/2	Bcl-2 family	Small-cell lung cancer
Terminated	A Study of AT-101 in Combination with Docetaxel in Squamous Cell Carcinoma of the Head and Neck	Phase 2	Bcl-2 family	Head and neck Squamous cell carcinoma
Completed	Safety & Efficacy Study of AT-101 in Combination w/ Rituximab in Previously Untreated Grade I–II Follicular Non-Hodgkin’s Lymphoma	Phase 2	Bcl-2 family	Follicular lymphoma
Completed	Phase II Safety and Efficacy Study of Single-agent AT-101 in Patients with Relapsed or Refractory B-cell Malignancies	Phase 2	Bcl-2 family	Lymphoma
Active, not recruiting	Chemotherapy and Bcl-xL Inhibitor (AT-101) for Organ Preservation in Adults with Advanced Laryngeal Cancer	Phase 2	Bcl-2 family	Laryngeal cancer
Completed	A Study of Single-Agent AT-101 in Men with Hormone Refractory Prostate Cancer	Phase1/2	Bcl-2 family	Prostate cancer

Completed or ongoing clinical trials using gossypol in cancer treatment and their reported mode of action were summarized using ClinicalTrials.gov (https://clinicaltrials.gov/). Data were downloaded on 5th September 2019. Bcl-2: B-cell lymphoma 2.

## Supplementary Materials

The following are available online at https://www.mdpi.com/2218-273X/9/10/595/s1, Figure S1: Molecular docking results of interaction between gossypol and potential target proteins.
