# Supplementary material for: Gossypol Suppresses Growth of Temozolomide-Resistant Glioblastoma Tumor Spheres
Source: Biomolecules. 2019 Oct 10;9(10):595. doi: 10.3390/biom9100595 (PMC6843396; doi:10.3390/biom9100595)
Supplement: Supplementary file 1 [file biomolecules-09-00595-s001.pdf]

SUPPLEMENTAL INFORMATION

Gossypol Suppresses Growth of Temozolomide-Resistant Glioblastoma Tumor Spheres

Hee Yeon Kim, Byung Il Lee, Ji Hoon Jeon, Dong Keon Kim, Seok-Gu Kang, Jin-Kyoung Shim, Soo Youl Kim, Sang Won Kang, and Hyonchol Jang

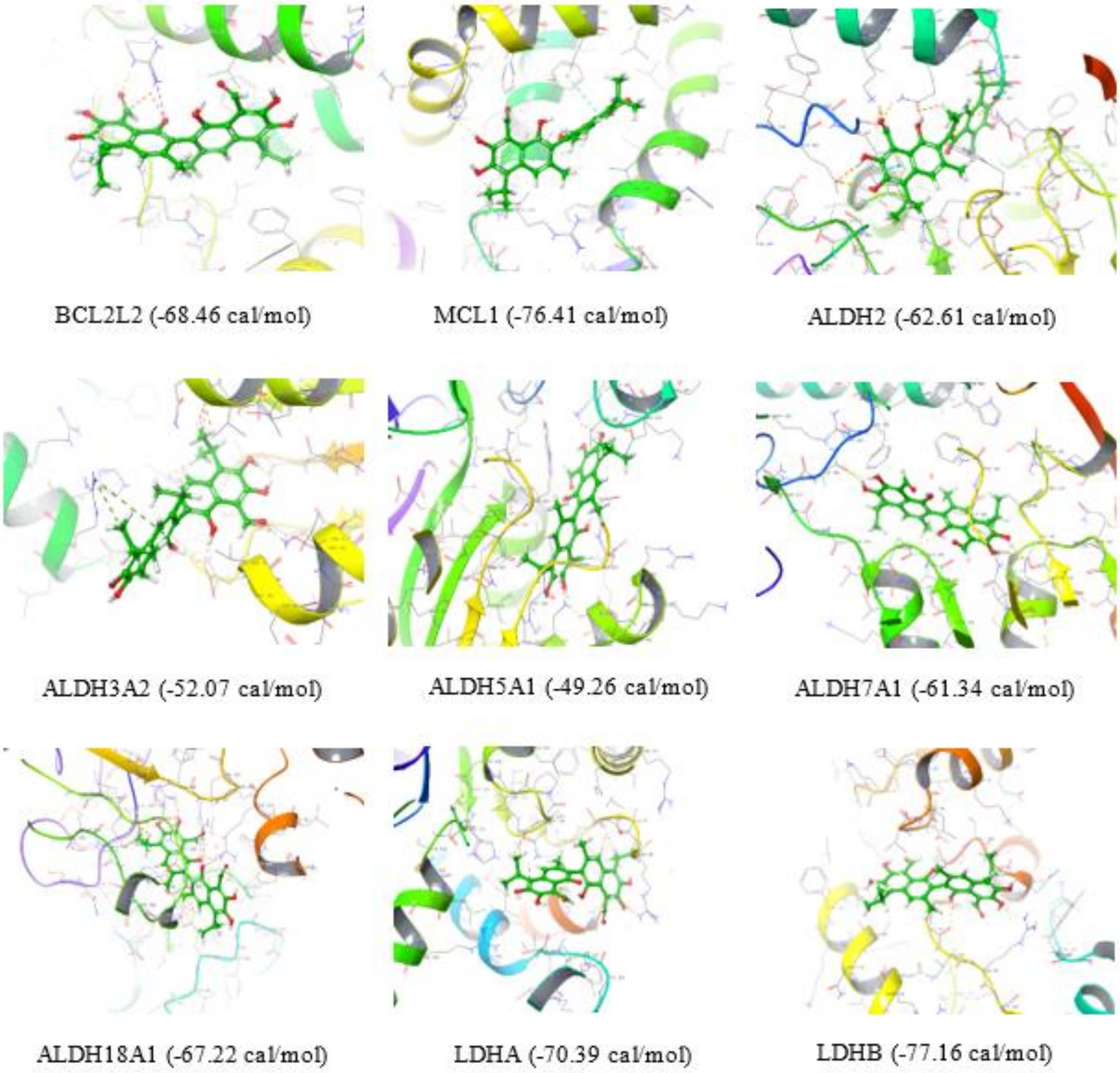

Figure S1. *Cont.*

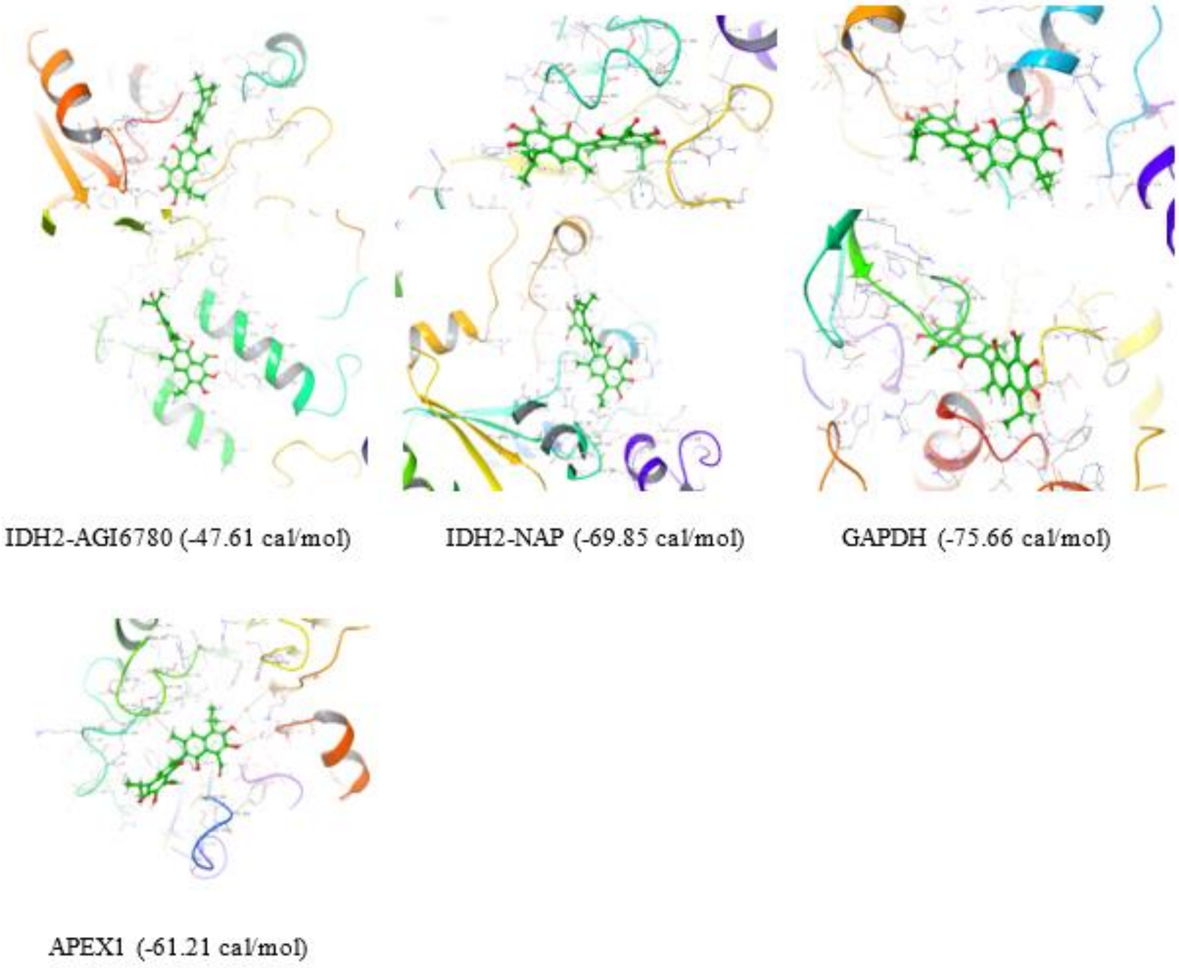

Figure S1. Molecular docking results of interaction between gossypol and potential target proteins. The binding energy by MM/GBSA method were presented. IDH1-59D and IDH2-AGI6780 are docking results targeting inhibitor binding sites of IDHs. IDH1-NAP and IDH2-NAP are docking results targeting enzyme's NADP binding sites.
